# Chlorophyll fluorescence, physiology, and yield of winter wheat under different irrigation and shade durations during the grain-filling stage

**DOI:** 10.3389/fpls.2024.1396929

**Published:** 2024-07-29

**Authors:** Muhammad Asad Naseer, Sadam Hussain, Ahmed Mukhtar, Qian Rui, Guo Ru, Haseeb Ahmad, Zhi Qin Zhang, Li Bo Shi, Muhammad Shoaib Asad, Xiaoli Chen, Xun Bo Zhou, Xiaolong Ren

**Affiliations:** ^1^ Guangxi Key Laboratory for Agro-Environment and Agro-Product Safety, Key Laboratory of Crop Cultivation and Physiology, College of Agriculture, Guangxi University, Nanning, China; ^2^ College of Agronomy, Northwest A&F University, Yangling, China; ^3^ Key Laboratory of Crop Physio-Ecology and Tillage Science in Northwestern Loess Plateau, Ministry of Agriculture, Northwest A&F University, Yangling, Shaanxi, China; ^4^ College of Horticulture, Northwest A&F University, Yangling, China; ^5^ Sinochem Modern Agriculture (Shandong) Co., Ltd, Jinan, China

**Keywords:** winter wheat, grain-filling, shading, drought, photochemistry, photosynthesis

## Abstract

The uneven spatial and temporal distribution of light resources and water scarcity during the grain-filling stage pose significant challenges for sustainable crop production, particularly in the arid areas of the Loess Plateau in Northwest China. This study aims to investigate the combined effects of drought and shading stress on winter wheat growth and its physio-biochemical and antioxidative responses. Wheat plants were subjected to different drought levels— full irrigation (I100), 75% of full irrigation (I75), 50% of full irrigation (I50), and 25% of full irrigation (I25), and shading treatments — 12, 9, 6, 3 and 0 days (SD12, SD9, SD6, SD3, and CK, respectively) during the grain-filling stage. The effects of drought and shading treatments reduced yield in descending order, with the most significant reductions observed in the SD12 and I25 treatments. These treatments decreased grain yield, spikes per plant, 1000-grain weight, and spikelets per spike by 160.67%, 248.13%, 28.22%, and 179.55%, respectively, compared to the CK. Furthermore, MDA content and antioxidant enzyme activities exhibited an ascending trend with reduced irrigation and longer shading durations. The highest values were recorded in the I75 and SD12 treatments, which increased MDA, SOD, POD, and CAT activities by 65.22, 66.79, 65.07 and 58.38%, respectively, compared to the CK. The Pn, E, Gs, and iCO_2_ exhibited a decreasing trend (318.14, 521.09, 908.77, and 90.85%) with increasing shading duration and decreasing irrigation amount. Drought and shading treatments damage leaf chlorophyll fluorescence, decreasing yield and related physiological and biochemical attributes.

## Introduction

Food security relies significantly on wheat production, the world’s most important cereal crop. In the loess Plateau of China, precipitation is the sole source of irrigation for winter wheat cultivation (Qiu et al., 2022). This region naturally experiences irregular and inadequate rainfall (Aixia et al., 2022). The annual rainfall ranges from 400-600 mm, with only 20-30% occurring during the winter wheat growth period, which is insufficient to meet the crop’s water requirements (Dong et al., 2019; Li et al., 2019). Furthermore, in China’s rainfed regions, the annual evaporation rate surpasses 830 mm, resulting in severe drought conditions throughout the entire growth period of winter wheat (Zhang et al., 2016). Among the developmental stages of winter wheat, the filling stage is most vulnerable to drought stress (Hlavacova et al., 2018; Hussain et al., 2019). In the Loess Plateau of China, inadequate light due to cloud cover during the grain-filling stage exacerbates this situation, leading to yield losses in maize (Naseer et al., 2023).

Compared to a single stress, co-occurring stressors can lead to differences in plants’ morphological and physiological responses. Plants subjected to both drought stress (55.2% field capacity) and low irradiance (PPFD = 500-600 mol m^-2^ s^-1^ at noon) did not exhibit a decrease in transpiration rate (E), stomatal conductance (gs), or net photosynthetic rate (Pn), unlike plants exposed to medium or high irradiance (Shafiq et al., 2020). This supports the facilitation hypothesis (Holmgren, 2000), suggesting that the level of irradiance in the environment impacts how drought stress affects plant’s photosynthetic ability. Furthermore, the presence of shade led to a reduction in the synthesis of reductants such as glutathione reductase, thioredoxin reductase, and ascorbate when drought stress and shade co-occurred (Ali et al., 2005; Baier et al., 2005; Ahmed et al., 2009). More substantial ROS-driven oxidative damage during drought is associated with reduced reduction ability (Fatemi et al., 2023). ROS damage leads to various physiological and metabolic abnormalities in plants (Hussain et al., 2019).

Light intensity plays a significant role in influencing various aspects of photosynthesis, including the rate of photosynthesis (Pn), transpiration rate, stomatal conductance, and light compensation and saturation points (Li et al., 2007; Ubierna et al., 2013). Chlorophyll fluorescence, which provides subtle insights into the primary reactions of photosynthesis, is a non-invasive tool used in ecophysiological studies to assess plant responses to environmental stress (Sommer et al., 2023). The intricate relationships between fluorescence kinetics and photosynthesis contribute to our understanding of the biophysical processes underlying photosynthesis. These processes also impact the composition of photosynthetic pigments, chloroplast structure, and Pn. Leaf adaptation to shading during development, particularly in chloroplasts, involves special biochemical adjustments. Under shade conditions, leaves contain more chlorophyll by weight but less per unit leaf area compared to leaves in full sun. Chloroplasts adapted for efficient photosynthetic quantum conversion have a higher photosynthetic capacity per leaf area and higher chlorophyll content, featuring elevated chlorophyll a and b values. Horie et al. (2006) demonstrated that canopy temperature is lower in shaded plants than in those exposed to full sun.

Leaves, as the primary photosynthetic organs, are significantly influenced by light levels. Plants’ capacity to adapt to suboptimal light conditions relies heavily on leaf characteristics (Li et al., 2010; Bande et al., 2013; Mauro et al., 2014). Leaf anatomy is impacted by light, but different species alter their leaf structure in varying ways (Pang et al., 2019). Relevant morphological changes include increased leaf area, decreased specific leaf weight (SLW), and a higher dry weight (DW) of leaves relative to stems or the total plant DW (Manoj et al., 2019; Angadi et al., 2022). Leaves developed under reduced sunlight are typically thinner but larger, resulting in a higher specific leaf area (SLA) (Rozendaal et al., 2006; Feng et al., 2008; Liu et al., 2016). These changes are likely adaptations to maximize light and carbon capture in low-light conditions, reducing the plant’s dry mass per unit leaf area and increasing the proportion of leaf biomass in the total plant biomass (Nurul Hafiza et al., 2014).

Our study utilized different shading intervals and irrigation gradients to quantify yield change under combined drought and shading conditions. The shading treatments correspond to the natural light/cloudy conditions during the grain-filling stage of winter wheat in the loess plateau of China. Meanwhile, the irrigation treatments correspond with the natural rainfall (mm) during the grain-filling stage. Our study aimed to (1) quantify the photochemistry of winter wheat flag leaves during the grain-filling stage, (2) assess the winter wheat yield reduction due to low light and drought conditions during the grain-filling stage, and (3) evaluate the changes in physiological parameters of winter wheat and their contribution to yield reduction under these combined stresses.

## Materials and methods

### Plant materials and experimental design

This experiment was conducted in a greenhouse at the Institute of Water Saving Agriculture Experimental Station of Northwest A&F University, Yangling (34°20′N, 108°24′E), China. The underground soil columns (with a diameter and length of 30 cm and 3 m, respectively) were filled with a mixture of farmland topsoil and compost in a 2:1 ratio (w/w). The study was conducted under waterproof sheds. The dimensions of the shed were 3 m (height) × 15 m (width) × 16 m (length). Moveable waterproof sheds were used to manage natural rainfall on rainy days. The experiment was conducted using a split-plot design with three replications.

### Crop management and radiation control

In this study, we used wheat (*Triticum aestivum* L.) cv. Xinong 979 which was obtained from Jun Hun Seed Company. Ten plants per column were physically harvested on May 29, 2022, after being manually planted on October 12, 2021. At the time of seeding, 225 mg kg^-1^ of nitrogen (from urea) and 75 mg kg^-1^ of phosphorus (from diammonium phosphate) were applied. Each soil column was irrigated with a precisely determined amount of water using pipes emerging from drums for irrigation application.

**Figure 1:**
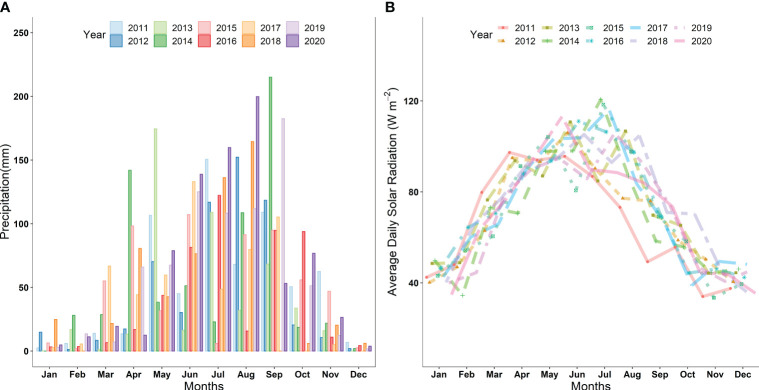
**(A)** Average monthly precipitation (mm) during 2011-2020 in the study area **(B)** Average daily solar radiation (Wm^-2^) during the growth period in 2011-2020.

The grain-filling stage, identified as Z70 (Zadoks et al., 1974), is particularly vulnerable to the impacts of drought and shading (Farooq et al., 2014; Hlavacova et al., 2018). Therefore, Z70 was selected as the treatment stage (Li et al., 2007; Mu et al., 2010). During the grain-filling stage of winter wheat, irrigation treatments were determined based on the region’s maximum and minimum historical rainfall values over the past 10 years. The maximum and minimum precipitation conditions for the area were identified as 168 mm and 0 mm, respectively (**Figure 1**). We divided these into 4 levels, corresponding to natural rainfall conditions: I100 indicates full irrigation (8.96 L), I75 represents 75% of full irrigation (6.72 L), I50 represents 50% of full irrigation (4.48 L), and I25 represents 25% (2.24 L) of total irrigation (8.96 L). These levels represent the percentage of total irrigation (8.96 L) applied during the grain-filling stage. Before the application of drought, the soil moisture in fixed underground columns was kept at 85–90% Field Capacity (FC). During the same period of irrigation treatments, with intervals of three days apart, five levels of shading treatment were applied: (1) SD12 (shading for 12 days), (2) SD9 (shading for 9 days), (3) SD6 (shading for 6 days), (4) SD3 (shading for 3 days), and (5) SD0 (0 days shading, CK). Shading was achieved using black plastic cover. A detachable shed measuring 12 meters long by 7 meters wide and with a height of 3.5 meters was constructed using scaffolding and black polypropylene fabric. The fabric extended 2 meters longer at the edge to block slanting sunlight. During the experiment, the photosynthetic photon flux density (PPFD) for the regular light treatment was about 150 ± 10 μmol photons m^−2^ s^−1^ and a red/far red (R:FR) ratio of 1.2. Under shading conditions, the PPFD was reduced to 75 ± 10 μmol photons m^−2 s−1^ with an R:FR ratio ranging from 0.4 ∼ 0.6.

### Sampling and measurements

#### Gas exchange parameters

The gas exchange parameters (rate of net photosynthesis, stomatal conductance, light intensity, and transpiration rate) were measured using a portable photosynthesis system LI- 6400XT (LI-COR, Biosciences, Lincoln, NE, USA). The CO_2_ concentration in the leaf chamber was maintained at 380 µmol mol^-1,^ and the photosynthetic active radiation was set at 1100 µmol m^-2^ s^-1^. Observations were recorded from the flag leaves between 9:00 to 11:00 AM after 12 days of shading duration during the grain-filling stage (12 days after Z70) (Urban et al., 2018) Three plants from each soil column were selected, and their flag leaves were tagged for these measurements.

#### Chlorophyll fluorescence measurements

Chlorophyll fluorescence was measured using Fluor Technologia software (Fluor Images, United Kingdom). Three fully expanded leaf samples from each column were collected and immediately preserved in plastic bags placed in an ice box to prevent exposed to direct light. The samples were then analyzed using a fluorescence analyzing device with the mentioned software. We examined the maximum quantum yield in the dark (Fv/Fm), quantum yield, photochemical quenching (qP), and non-photochemical quenching (NPQ) using the FluorImager software, Technologia LTD (Hussain et al., 2019).

### Grain yield and yield components

Twenty spikes per column were harvested at maturity and threshed to separate the grains from the straw. The number of kernels per spike was counted, and 1000 grains were counted and weighed. Five tillers were randomly selected in each soil column to measure plant height using a meter rod. Spike length (distance from the base to the end of the spike) was measured with a ruler. Additionally, three plants were randomly selected from each column to record the grain yield.

### Malondialdehyde contents and antioxidant enzyme activities

Three flag leaf samples from each column were taken and preserved in liquid nitrogen after 12 days of shading treatment. These samples were stored in the refrigerator at -80°C. Leaf malondialdehyde (MDA) contents, an index of lipid peroxidation, were determined using the method described by (Cakmak and Marschner, 1992) with slight modifications. 500 mL of supernatant from the MDA reaction mixture (containing 0.65% (w/v) thiobarbituric acid in 20% trichloroacetic acid) was heated for 30 min and then quickly chilled to halt the reaction. The mixture was then centrifuged at 10,000*g* for 10 min. The absorbance of the mixture was measured at 532 nm, and non-specific absorption was accounted for by subtracting the absorbance at 600 nm.

For the determination of superoxide dismutase (SOD), peroxidase (POD), and catalase (CAT) activities, 0.2 g frozen leaf tissues were ground in 5 mL of 0.1 mol L^–1^ Tris-HCl buffer (pH 7.8) containing 1% polyvinyl pyrrolidone, 1 mmol L^–1^ EDTA, and 1 mmol L^–1^ dithiothreitol. The homogenate was centrifuged at 18000*g* for 20 min at 4°C. The supernatant was subsequently used to measure enzyme activities.

For the determination of SOD activity, the reaction mixture contained 0.2 mL of the enzyme solution mixed with 50 mM phosphate buffer (pH 7.6), 13 mM methionine, 750 mM NBT, 4 mM riboflavin, and 0.1 mM EDTA. The photochemical reduction of NBT was measured following the procedure of Lei et al. (2006). Catalase activity was assayed by mixing the reaction mixture containing 50 mM phosphate buffer (pH 7.0) and 12.5 mM H_2_O_2_ with enzyme extract, following the method of (Djanaguiraman et al., 2009). To estimate POD activity, 50 mM phosphate buffer (pH 7.0), 16 mM guaiacol, enzyme extract, and 10 mM H_2_O_2_ were added to the reaction mixture. The POD activity was determined as described by Cakmak and Marschner (1992).

### Statistical analysis

Data were analyzed using two-way analysis of variance (ANOVA) to assess the effects of drought and shading treatments. This analysis was conducted using R-software (Version; 4.1.0) with the support of the agricolae package (Version 1.3-5) to confirm variability. The Tukey HSD test was used to quantify differences between treatments at a 5% probability level. Data representation and illustration were performed using Origin software. Pearson correlation analysis was conducted using the pandas package (cluster map) in Python 3.12 to examine relationships among the studied parameters.

## Results

### Effect of shading and drought stress on gas exchange parameters

**Figure 2:**
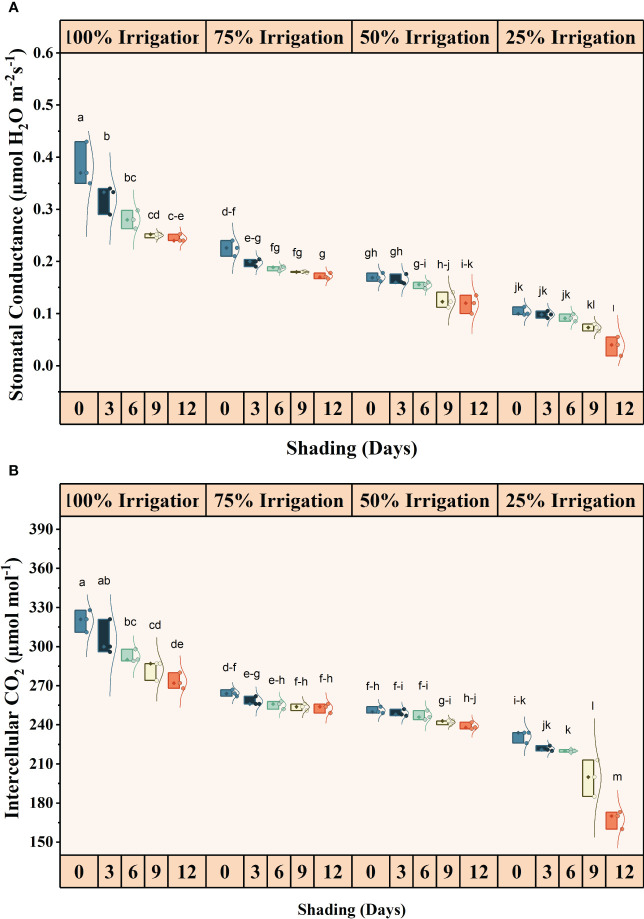
Effect of shading durations on **(A)** stomatal conductance and **(B)** intracellular CO_2_ concentration of wheat under different irrigation conditions (100, 75, 50 and 25% irrigation). The values represent the mean ± standard error, and bars sharing similar letters for a parameter indicate non-significant (p<0.05) differences.

**Figure 3:**
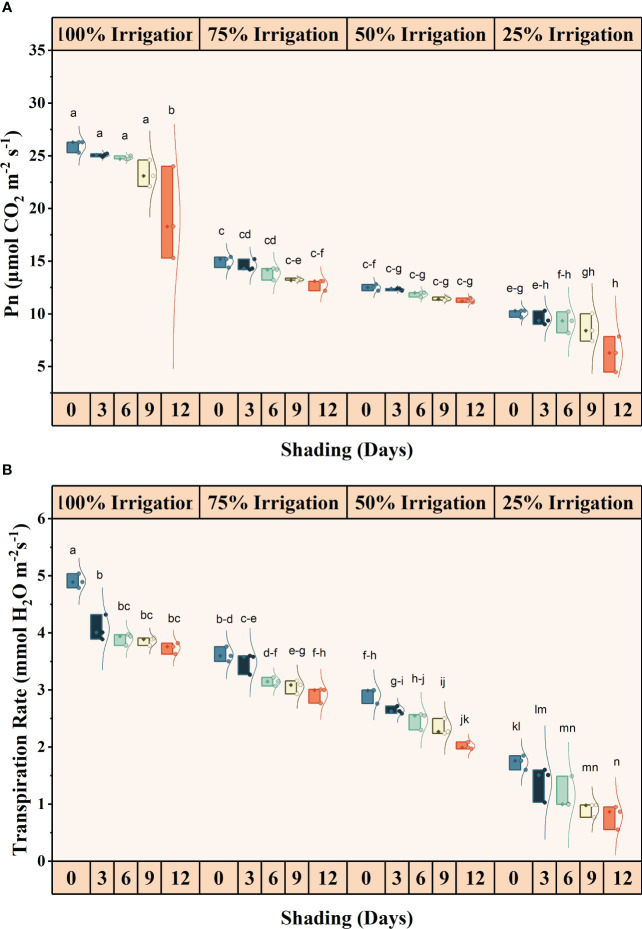
Effect of shading duration on **(A)** photosynthesis, and **(B)** transpiration rate of wheat under different irrigation conditions (100, 75, 50 and 25% irrigation). The values represent the mean ± standard error, and bars sharing similar letters for a parameter indicate non-significant (p<0.05) differences.

The irrigation and shading treatments significantly affected the photosynthetic activity (Pn), transpiration rates (E), stomatal conductance (Gs), and intercellular CO_2_ concentrations (iCO_2_) (**Supplementary Table S1**). The interactive influence was also significant for these traits (**Figures 2**, **3**). Pn, E, Gs, and iCO_2_ showed a decreasing trend with increasing shading duration and decreasing irrigation amount, with the lowest values observed under conditions of high-duration shading and minimum irrigation supply conditions (I25). Shading for 12 days and 75% irrigation reduction demonstrated a significant decrease of 318.14, 521.09, 908.77, and 90.85% in Pn, E, Gs and iCO_2_, respectively, as compared with no shading and full irrigation.

### Effect of shading and drought stress on chlorophyll fluorescence

**Figure 4:**
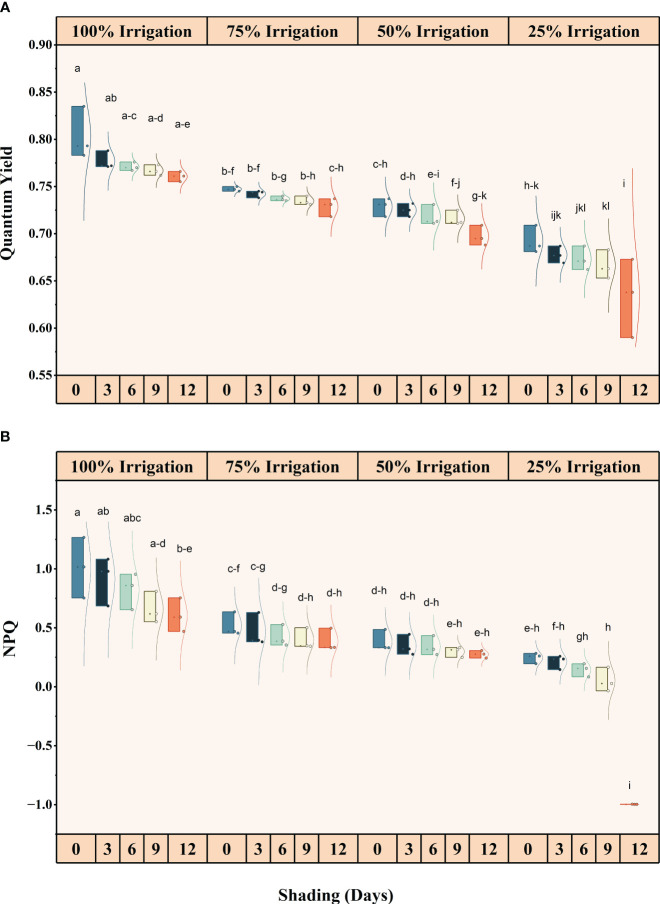
Effect of shading durations on **(A)** quantum yield, and **(B)** Non-Photochemical quenching (NPQ) of wheat leaves under different irrigation conditions (100, 75, 50 and 25% irrigation). The values represent the mean ± standard error, and bars sharing similar letters for a parameter indicate non-significant (P<0.05) differences.

**Figure 5:**
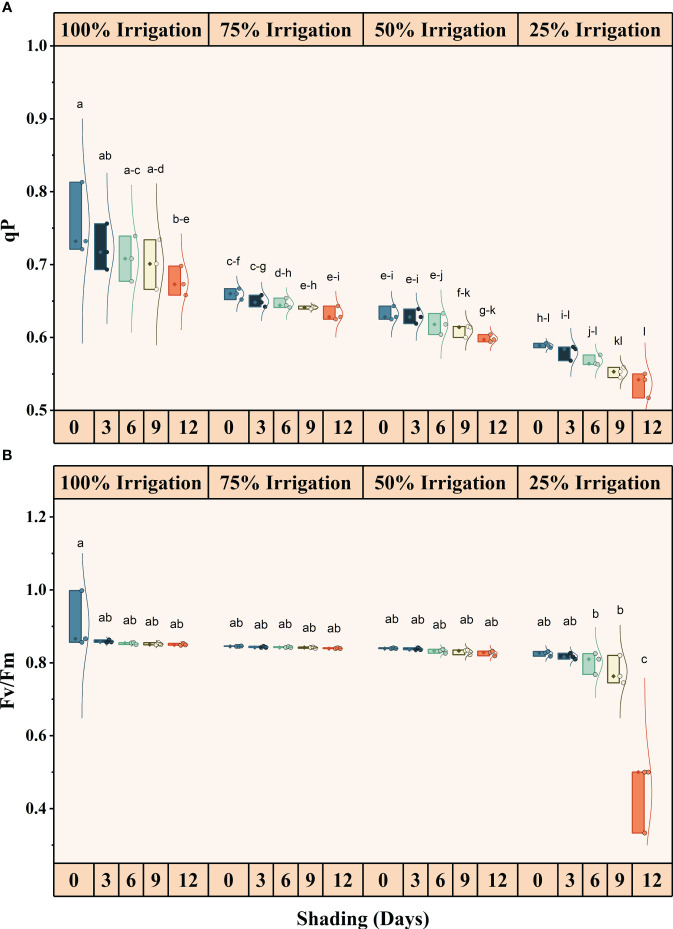
Effect of shading durations on **(A)** Photochemical quenching, and **(B)** Fv/Fm of wheat leaves under different irrigation conditions (100, 75, 50 and 25% irrigation). The values represent the mean ± standard error, and bars sharing similar letters for a parameter indicate non-significant (P<0.05) differences.

Irrigation intervals and shading duration, both individually and interactively, significantly (*P<*0.05) influenced chlorophyll fluorescence (**Supplementary Table S1**) (**Figures 4**, **5**). The quantum yield, qP, NPQ, and Fv/Fm decreased in descending order with increasing irrigation intervals and shading duration. The maximum reduction in these traits was recorded under shading for 12 days and 25% irrigation, with reductions of approximately 26.82%, 40.83%, 201.56%, and 105.05% in quantum yield, qP, NPQ, and Fv/Fm, respectively, compared to the full irrigation and no shading treatment.

### Effect of shading and drought stress during grain filling on yield and yield parameters of winter wheat

The stress treatments had a significant effect, i.e., irrigation intervals, shading durations, and their interactions, on yield and yield-related traits. These traits decreased in descending order with increasing irrigation interval and shading duration (**Table 1**). The maximum reduction in these traits was recorded in plants exposed to shading for 12 days and supplemented with only 25% irrigation, with reductions of 160.67% in spikes per plant, 248.13% in spikelets per spike, 28.22% in 1000-grains weight and 179.55% in grain yield, compared to the full irrigation and no shading treatment (**Table 1**).

**Table 1:** Effect of shading durations on spikes per plant, grains per spike, 1000-grain weight, and grain yield of winter wheat under different irrigation regimes.

Irrigation regimes (IR)	Shading durations (SD) (days)	Spikes per plant (number)	Grains per spike (number)	1000 grain weight (g)	Grain yield
100	12	19.55 ± 0.69c-h	19.33 ± 2.52c-e	42.53 ± 0.27c-e	16.1 ± 0.351b-e
	9	21.32 ± 0.87c-f	23.00 ± 2.65b-d	42.95 ± 0.07c-e	17.2 ± 1.05a-c
	6	23.09 ± 0.43bc	25.00 ± 2.00a-c	43.68 ± 0.29bc	16.86667 ± 0.81a-d
	3	27.70 ± 3.16b	29.00 ± 2.65a	46.41 ± 0.36ab	18.43667 ± 0.42ab
	0	34.20 ± 3.54a	28.67 ± 2.52ab	47.06 ± 0.28a	19.01667 ± 0.34a
75	12	19.88 ± 1.27c-h	11.33 ± 1.15gh	40.28 ± 0.36e-g	14.14333 ± 1.48ef
	9	21.82 ± 0.73c-e	13.00 ± 2.0f-h	40.89 ± 0.20def	15.77333 ± 1.51bcde
	6	24.03 ± 1.82bc	15.67 ± 1.15e-g	41.29 ± 0.05c-f	14.30333 ± 0.92def
	3	27.90 ± 1.02b	17.67 ± 1.15d-f	42.07 ± 0.12c-f	15.81333 ± 1.34bcde
	0	35.66 ± 5.50a	18.00 ± 1.73d-f	42.34 ± 0.01c-e	15.80667 ± 0.65bcde
50	12	13.52 ± 0.13i	11.33 ± 0.58gh	40.35 ± 1.52e-g	9.733333 ± 0.06gh
	9	15.02 ± 0.55g-i	13.33 ± 0.58f-h	37.95 ± 0.06gh	13.73333 ± 0.47f
	6	16.14 ± 0.04e-i	8.67 ± 1.53h	41.55 ± 2.54c-f	12.26 ± 1.49fg
	3	19.67 ± 2.44c-h	13.67 ± 1.53e-h	43.58 ± 1.00cd	12.4 ± 1.3fg
	0	22.54 ± 0.37b-d	13.00 ± 2.65f-h	46.43 ± 1.00ab	14.74333 ± 0.56cdef
25	12	13.12 ± 0.63i	8.33 ± 1.15h	36.70 ± 1.23h	6.8 ± 0.62ij
	9	14.19 ± 0.50hi	12.67 ± 1.15f-h	37.31 ± 1.16h	7.526667 ± 0.67hij
	6	15.78 ± 0.44f-i	10.67 ± 1.53gh	37.69 ± 1.13gh	6.533333 ± 0.15j
	3	17.09 ± 0.77d-i	14.67 ± 1.15e-g	37.25 ± 0.05h	8.466667 ± 0.40hij
	0	20.64 ± 1.37c-g	15.33 ± 3.79e-g	39.45 ± 0.85f-h	9.45 ± 0.13hi
Analysis of variance					
LSD (*p*< 0.05)		Spikes/plant	Kernels/spike	1000-grain weight	Grain yield
IR		(***) <2 × 10^-16^	(***) <2 × 10^-16^	(***) <2 × 10^-16^	(***) <2 × 10^-16^
SD		(***) <2 × 10^-16^	(***) 8.67 × 10^-10^	(***)6.06 × 10^-14^	(***) 1.04 × 10^-9^
IR×SD		(*)0.018	(*) 0.0134	(***)6.35 × 10^-6^	(*) 0.048

IR, irrigation regimes; SD, shading durations (days); (***), p < 0.001; (**), p< 0.01; (*), p< 0.05; (ns), non-significant. Values represent means ± standard error. Means sharing similar letters for a parameter indicates non-significant (P<0.05) differences.

### Effect of shading and drought stress on antioxidants and malondialdehyde contents

**Figure 6:**
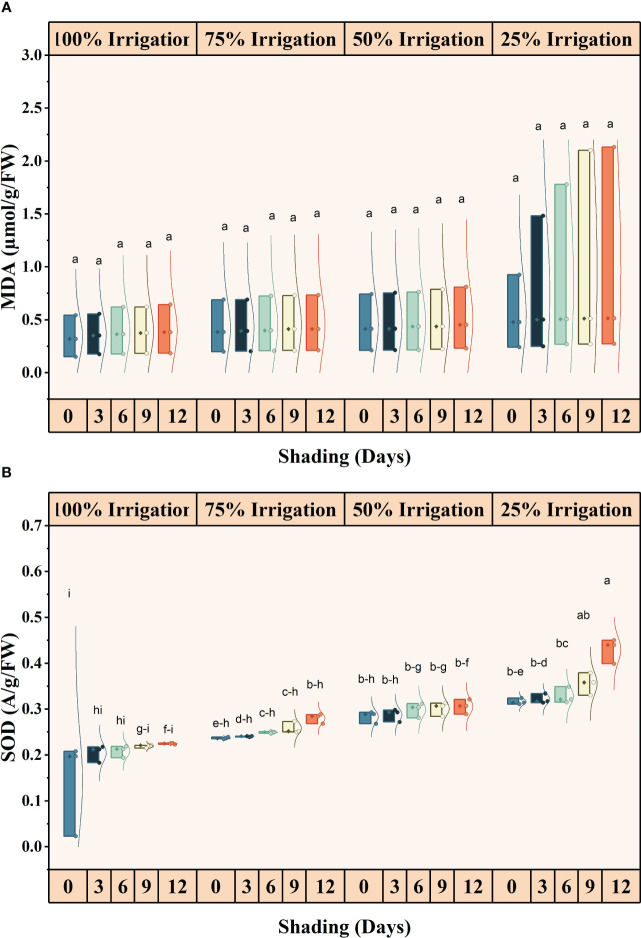
Effect of shading durations on **(A)** malondialdehyde (MDA) and **(B)** superoxide dismutase (SOD) of wheat under different irrigation conditions (100, 75, 50 and 25% irrigation). The values represent the mean ± standard error, and bars sharing similar letters for a parameter indicate non-significant (P<0.05) differences.

**Figure 7:**
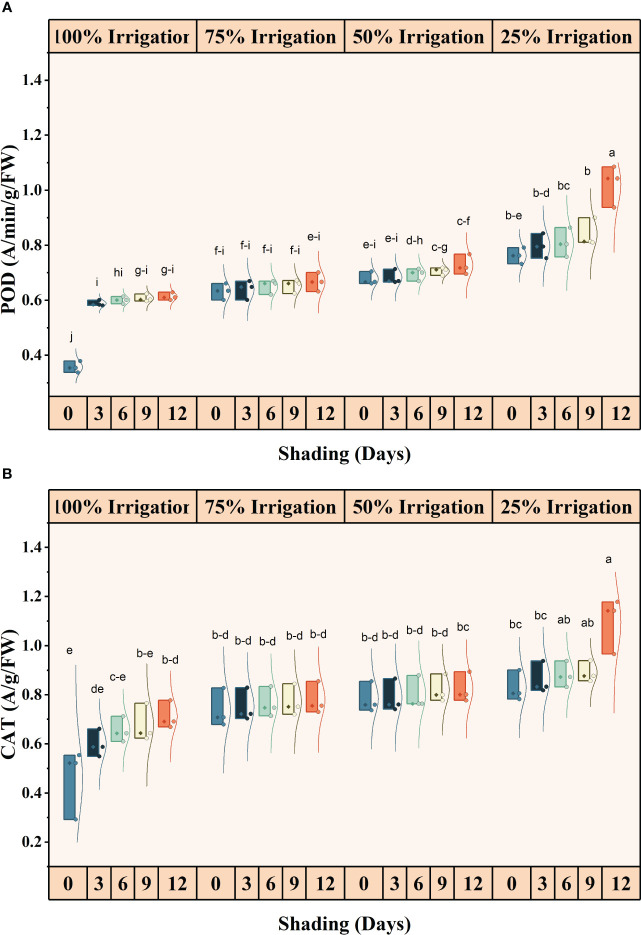
Effect of shading durations on **(A)** peroxidase (POD) and **(B)** catalase (CAT) of wheat under different irrigation conditions (100, 75, 50 and 25% irrigation). The values represent the mean ± standard error, and bars sharing similar letters for a parameter indicate non-significant (P<0.05) differences.

The antioxidant activities (SOD, POD and CAT) were significantly different for shading duration (SD), irrigation and combined shading and irrigation (SD×I) treatments (**Figures 6**, **7**). SOD, POD, and CAT activities, increased with reduced irrigation amounts and longer shading durations. Compared with full irrigation and no shading treatment, a 75% reduction in irrigation and 12 days of shading increased SOD, POD, and CAT activity by 66.79, 65.07 and 58.38%, respectively. However, the increase in MDA contents was not significant for IR, SD, and IR×SD treatments (**Supplementary Table S1**).

### Correlation analysis and principal component analysis

**Figure 8:**
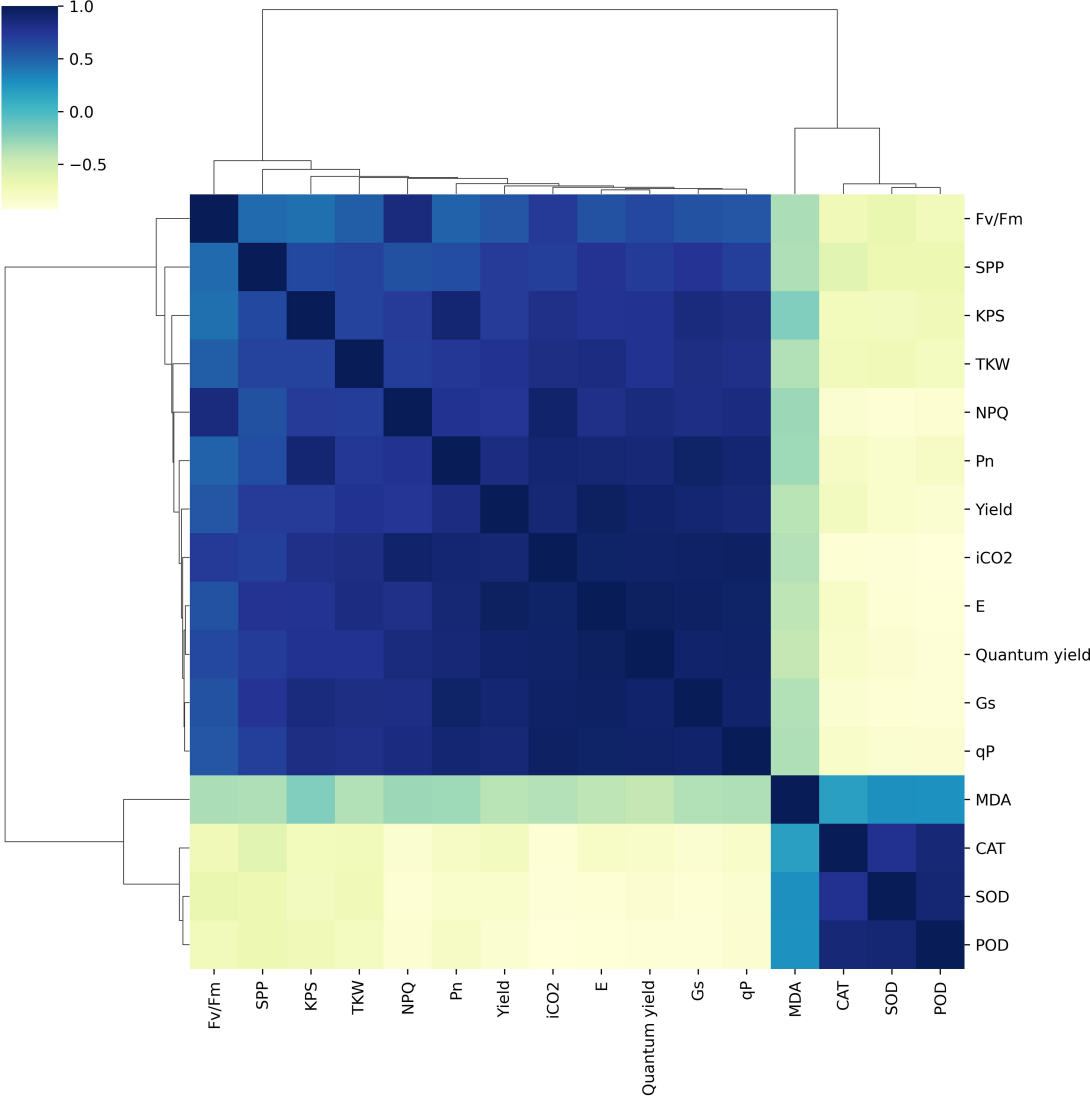
Relationships among net photosynthetic rate, antioxidant enzymes, chlorophyll fluorescence, lipid peroxidation, and grain yield. Pn, photosynthetic activity; E, transpiration rate; iCO2, intracellular CO_2_ concentration; Gs, stomatal conductance; SOD, superoxide dismutase; CAT, catalase; POD, peroxidase; MDA, malondialdehyde; Yield, grain yield; SPP, spikes per plant; KPS, Kernels per spike; TKW, thousand kernel weight.

The chlorophyll fluorescence, gas exchange parameters, antioxidant activities, and yield traits were significantly correlated under irrigation and shading treatments (**Figure 8**). Pn had a strong positive correlation with iCO_2_, Gs, E, and yield traits (SPP, KPS, TKW and yield) while showing a strong negative correlation with antioxidant activities. Likewise, iCO_2_, Gs, and E were strongly positively correlated with each other, as well as with chlorophyll fluorescence, gas exchange, and yield traits, but had a strong negative correlation with antioxidant activities. SOD showed a strong positive correlation with POD and CAT, while exhibiting a strong negative correlation with gas exchange traits, chlorophyll fluorescence, and yield-related traits.

**Figure 9:**
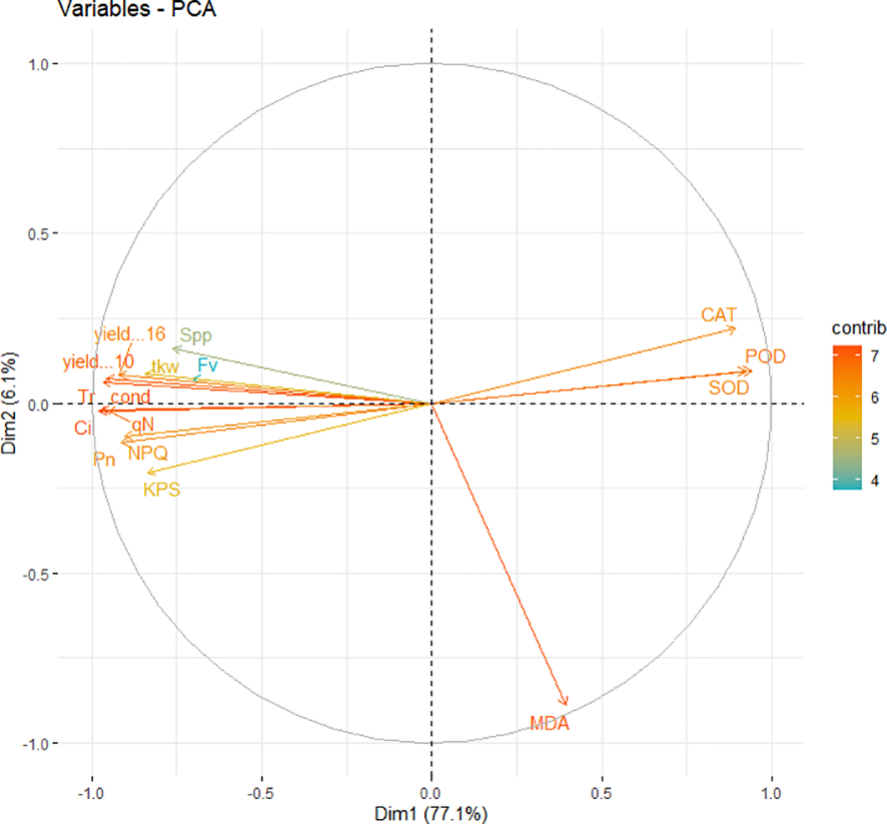
PCA (principal component analysis) of photosynthetic activity, chlorophyll fluorescence, malondialdehyde contents, antioxidants enzymes and yield parameters.

**Figure 10:**
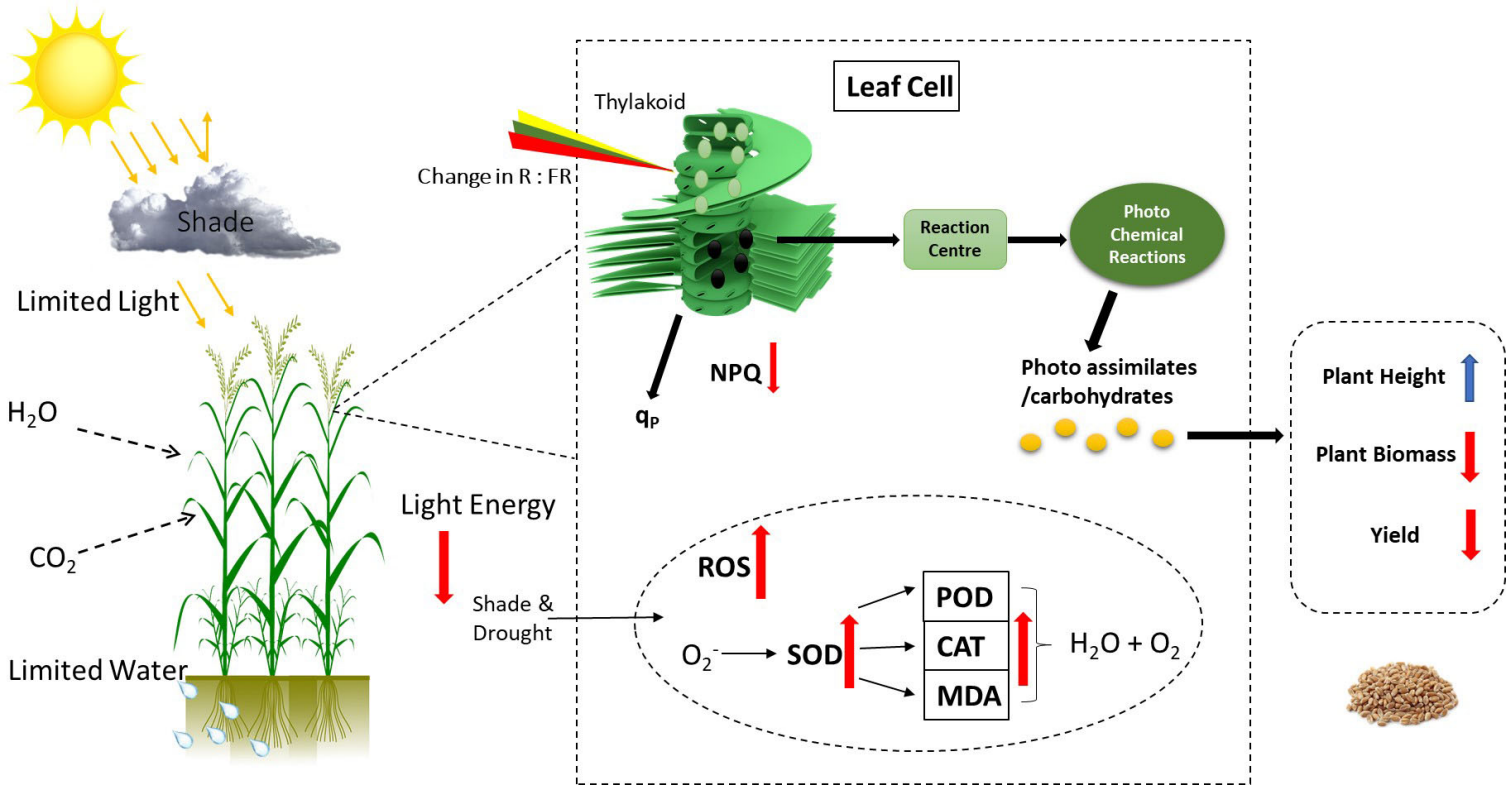
The comprehensive model of physiological metabolism regulation in winter wheat plants under drought and shading stress. Changing the light environment and drought conditions regulate the photosynthetic activity, photochemical efficacy, and antioxidant enzyme activities to adapt the environmental stress. The distribution and regulation of photo-assimilates affect the agronomic characteristics, and yield of winter wheat plants.

Overall, gas exchange traits, chlorophyll fluorescence, and yield-related traits had a significantly strong correlation with each other. Furthermore, principal component analysis was conducted using recorded data on gas exchange, photosynthetic traits, and yield attributes. It was noted that PC1 captured about 77.1% of the inertia of the data and was strongly related to CAT, SOD, and POD activity (**Figure 9**), indicating that antioxidant activities accounted for seedlings’ responses to irrigation and shading treatments. PC2 described only 6.1% of the variance and was mainly determined by gas exchange and chlorophyll fluorescence. The comprehensive model of the change in photosynthetic activity, antioxidant activities, photochemical efficiency and yield due to combined effect of shading and drought on winter wheat is shown in (**Figure 10**).

## Discussion

### Gas exchange and photochemical reactions

Consistent with previously published studies, we observed a significant reduction in quantum yield, Fv/Fm, qP and NPQ under different shading durations as drought stress severity increased (Hussain et al., 2019a). Due to its sensitivity and utility, chlorophyll fluorescence is a crucial indicator of photosynthetic efficiency and plant responses to environmental variables (Dai et al., 2009). Reduced electron flow through PSII is typically associated with decreased photosynthetic capacity (Yao et al., 2017a). Previous studies have shown that crops grown in shaded conditions (Hussain et al., 2019a; Hussain et al., 2019b) as well as under drought stress tend to exhibit lower values of quantum yield, effective quantum yield of photosystem (PSII), photochemical quenching (qP), and electron transport rate (ETR) (Mafakheri et al., 2010; Abid et al., 2017; Mathobo et al., 2017).

According to the findings, the impact on the photosynthetic electron transport chain and leaf water loss reduced the photorespiration rate as shade intensity or duration decreased. Notably, the changes in Pn and Gs were closely linked to the light level (Yao et al., 2017b). Conversely, the photosynthesis rate of shaded leaves decreased due to the reduced solar radiation and increased diffuse light (Ping et al., 2015). Previous reports indicate that stomatal limitation is the primary factor causing lower photosynthesis during drought (Silva et al., 2013).

Results showed that limited irrigation decreased the maximum photochemical efficiency of PSII (Fv/Fm), the probability of electron transport beyond QA (1-VJ), and the ratio of (1-VI)/(1-VJ), which express the efficiency with which an electron from the intersystem electron carriers moves to electron acceptors at the PSI acceptor side. This reduction was more pronounced under the control treatment (no shade) than other treatments (**Figure 5**). Due to its sensitivity to stress, chlorophyll fluorescence can reliably represent changes in photosynthesis under drought and shade stress. According to Naramoto et al. (2006), protein phosphatases are thought to dephosphorylate LHCII (the light-harvesting chlorophyll protein) in situations of decreasing light intensity (shading conditions), causing the mobile light receptor antennae to revert to PSII (Naramoto et al., 2006; Strasser et al., 2010). Due to the overstimulation of PSII, leading to a shift in the mobile antennae, the efficiency of total electron transport is higher in shaded conditions than in full sunshine. Under field conditions, where plants typically experience both water stress and high light levels, down-regulated photosynthesis occurs due to the interaction between water stress and excessive light (Rakic et al., 2015). Light enhances evaporation and dehydrates the leaves, and it can also directly induce photoinhibition, which is the temporary damage to proteins in the photosynthesis reaction centers (Yamazaki et al., 2011). Both evaporation and photoinhibition can reduce plant photosynthetic activity. In an experiment, Li and Ma (2012) found that direct photoinhibition of light on dehydrated apple tree leaves was the primary cause of decreased PSII activity.

### Antioxidant enzyme activity and ROS generation

Drought and shade conditions cause oxidative stress in plants, leading to increased ROS generation and inducing lipid peroxidation. This process damages the plant’s cell membrane and results in oxidative damage to DNA, protein, and chlorophyll pigments, ultimately causing cell death (Naseer et al., 2022). In response to oxidative stress, plants produce a complex array of antioxidant enzymes such as SOD, POD, and CAT. These enzymes prevent uncontrolled oxidation by ROS and maintain a balance between ROS production and removal, which is essential for the optimal functioning of photosynthesis (Foyer, 2018). We found that enzymatic activity was substantially higher in shaded conditions compared to full light. Additionally, increasing auxin levels under simultaneous shade and drought stress enhanced antioxidant enzymatic activity (Duan et al., 2005). Moreover, as drought stress intensifies, plants under shade stress increase their synthesis of antioxidants to control their redox balance, thus mitigating the severe consequences of drought stress (Asghar et al., 2020).

### Partitioning of absorbed light energy and photorespiration

Our results illustrate a reduction in the quantum yield of PSII under limited irrigation compared to full irrigation and across all shading treatments, as well as a decrease in the capture efficiency of excitation energy (Fv/Fm) (**Figure 5**). Notably, when shade was provided throughout the entire growing season, as opposed to previous shading treatments, the values of quantum yield and Fv/Fm exhibited a significant decrease (**Figure 5**). This decrease is likely attributed to the longer duration of shade exposure and lower irrigation levels. Furthermore, the non-photochemical quenching (NPQ) experienced a significant decrease due to the combination of shade and water stress (**Figure 4**). The values for Fv/Fm and quantum yield were higher in the control treatment (no shade) when plants received full irrigation. However, NPQ was still greater in the shading treatment, even under full irrigation conditions. As longer shade durations (SD12) were imposed, changes in the photorespiration rate suggested that more photosynthetic electrons were partitioned to photorespiration during water deficiency stress. Only a minimal amount of light energy is used for photosynthesis during drought stress (closed stomata and subsequent secondary light stress due to a lack of CO_2_), and nearly all of the available energy must be securely disposed of. Photorespiration can sustain the Calvin cycle when CO_2_ availability restricts photosynthesis by making phosphoglycerate available (Suorsa and Aro, 2007). Nonetheless, it should be noted that the rate of leaf water loss was the main factor controlling photorespiration in stressed plants (Corpas et al., 2001).

The reduced stomatal density, leaf thickness, cross-sectional size of the vascular bundle, and contact area of the bundle sheath cells (Baldi et al., 2012) may contribute to reduced photosynthetic capability under shading conditions (Sultan, 2000). Modifications in leaf anatomy, morphology, physiology, and function can decrease photosynthesis. The physiology of leaves responds to shade in two ways: lower canopy leaves may age rapidly in intense shade conditions before the whole plant undergoes monocarpic senescence. Alternatively, another response involves the adaptation of photosynthesis in shaded leaves that persist on the plant until monocarpic senescence.

### The facilitative effect of shading under drought conditions

Interestingly, some studies have also reported that under shaded conditions, as opposed to full light, the rate of Pn increased significantly. This findings indicate a beneficial effect of shade under drought conditions, supporting the facilitation theory (Holmgren, 2000; Quero et al., 2006). Several processes may contribute to the facilitative impact of shade under drought stress. First, in light-limited conditions, the sensitivity of gs to drought was reduced, suggesting that the stomatal inhibition caused by drought was lessened (Prider and Facelli, 2004). Secondly, as indicated by reduced Fv/Fm, drought led to moderate photo-inhibitory injury in the photosystem II of plants grown in full-light conditions. This result suggests that the amount of light absorbed by plants exceeded what was necessary for photosynthesis, a condition exacerbated by drought (Demmig-Adams and Adams, 1992). Finally, the shade-induced rise in Pn became more favorable when the water supply decreased. This phenomenon is attributed to the lower air temperature in shaded conditions, which reduces the demand for water for transpiration. Consequently, plants can store more water and maintain a healthier tissue water status (Valladares and Pearcy, 1997; Prider and Facelli, 2004). These observations, consistent with previous studies, likely elucidate the positive impact of the drought-shade interaction on biomass production by mitigating the adverse effects of drought.

## Conclusions

Shading and drought stress significantly affected winter wheat’s physiological, biochemical, and yield traits. Both drought and shading treatments caused a marked decrease in yield and related traits, with a positive correlation between yield and associated traits such as spikes per plant, grains per spike, and 1000-grain weight. Furthermore, shading and drought affected physiological and biochemical characteristics, with values decreasing values as stress intensity increased. These reductions in physiological and biochemical traits ultimately led to a substantial decrease in winter wheat yield. Shading is a common abiotic stress in crop cultivation, significantly impacting crop productivity. Unfortunately, this stress has often been overlooked, despite its detrimental effects on crop growth, especially in intercropping systems and high-density monocropping systems, where crops frequently encounter shade throughout their lifespan. Plants employ numerous intricate biochemical, physiological, and molecular mechanisms to adapt to shade stress. Recent advancements in biotechnology have been instrumental in elucidating how plants respond to shade stress. However, further research is needed to fully explore these techniques. Identifying essential genes, proteins, metabolites, and other factors is possible using contemporary computational and systems biology technologies.

## Data availability statement

The original contributions presented in the study are included in the article/**Supplementary Material**, further inquiries can be directed to the corresponding author/s.

## Author contributions

MN: Conceptualization, Data curation, Formal analysis, Methodology, Software, Writing – original draft, Writing – review & editing. SH: Validation, Writing – review & editing. AM: Writing – review & editing. QR: Writing – review & editing. GR: Writing – review & editing. HA: Writing – review & editing. ZZ: Writing – review & editing. LS: Writing – review & editing. MA: Writing – review & editing. XC: Conceptualization, Supervision, Writing – review & editing. XZ: Funding acquisition, Supervision, Writing – review & editing. XR: Funding acquisition, Supervision, Validation, Writing – review & editing.
