# Supplementary material for: Chlorophyll fluorescence, physiology, and yield of winter wheat under different irrigation and shade durations during the grain-filling stage
Source: Front Plant Sci. 2024 Jul 29;15:1396929. doi: 10.3389/fpls.2024.1396929 (PMC11317437; doi:10.3389/fpls.2024.1396929)
Supplement: Supplementary file 1 [file Table_1.docx]

**Table S1:** Effect of shading duration (SD), irrigation regimes (IR), and their interaction (IR×SD) on different parameters recorded.

| **Measurements** | **IR** | **SD** | **IR×SD** |
| --- | --- | --- | --- |
| Pn | <2 × 10^-16^ *** | 9.13 × 10^-08^ *** | 0.0441 * |
| iCO_2_ | <2 × 10^-16^ *** | 9.01 × 10^-16^ *** | 1.16 × 10^-08^ *** |
| Gs | <2 × 10^-16^ *** | 3.46 × 10^-15^ *** | 1.55 × 10^-05^ *** |
| E | <2 × 10^-16^ *** | <2 × 10^-16^ *** | 0.0562 (ns) |
| SOD | <2 × 10^-16^ *** | 1.33 × 10^-05^ *** | 0.0806 (ns) |
| POD | <2 × 10^-16^ *** | 1.46 × 10^-11^ *** | 1.37 × 10^-08^ *** |
| CAT | 2.06 × 10^-12^ *** | 0.000223 *** | 0.078158 ns |
| MDA | 0.0525 (ns) | 0.9421 (ns) | 1.0000 (ns) |
| Fv/Fm | 3.46 × 10^-6^ *** | 0.000237 *** | 0.000428 *** |
| Quantum yield | <2 × 10^-16^ *** | 4.36 × 10^-8^ *** | 0.305 (ns) |
| qP | < 2 × 10^-16^ *** | 2.29 × 10^-8^ *** | 0.46 (ns) |
| NPQ | 4.61 × 10^-15^ *** | 1.82e × 10^-6^ *** | 0.0128 * |
|  |  |  |  |
| LSD (*p*< 0.05) | | | |

Note: IR, irrigation regimes; SD, shading days; (***), p<0.001; (**), p<0.01; (*), p<0.05; (ns), non-significant. Values represent means ± standard error.
